# The Effects of Sodium Tetraborate against Lead Toxicity in Rats: The Behavior of Some Metabolic Enzymes

**DOI:** 10.1021/acsomega.3c01021

**Published:** 2023-04-11

**Authors:** Mahire Bayramoğlu Akkoyun, Yusuf Temel, H.Turan Akkoyun, Şule Melek, Fatma Karagözoğlu, A. Şükrü Bengü, Kübra Geçmez

**Affiliations:** † Faculty of Veterinary Science, Department of Biochemistry, Siirt University, 56100, Siirt, Turkey; ‡ Solhan Health Services Vocational School, Bingol University, 12000, Bingol, Turkey; § Department of Surgery, Faculty of Veterinary Science, Bingol Universıty, 12000, Bingöl, Turkey; ⊥ Vocational School of Health Services, Bingöl University, 12000, Bingöl, Turkey; ∥ Faculty of Veterinary Science, Department of Animal Nutrition, Dokuz Eylul Universitesi, 35890, İzmir, Turkey

## Abstract

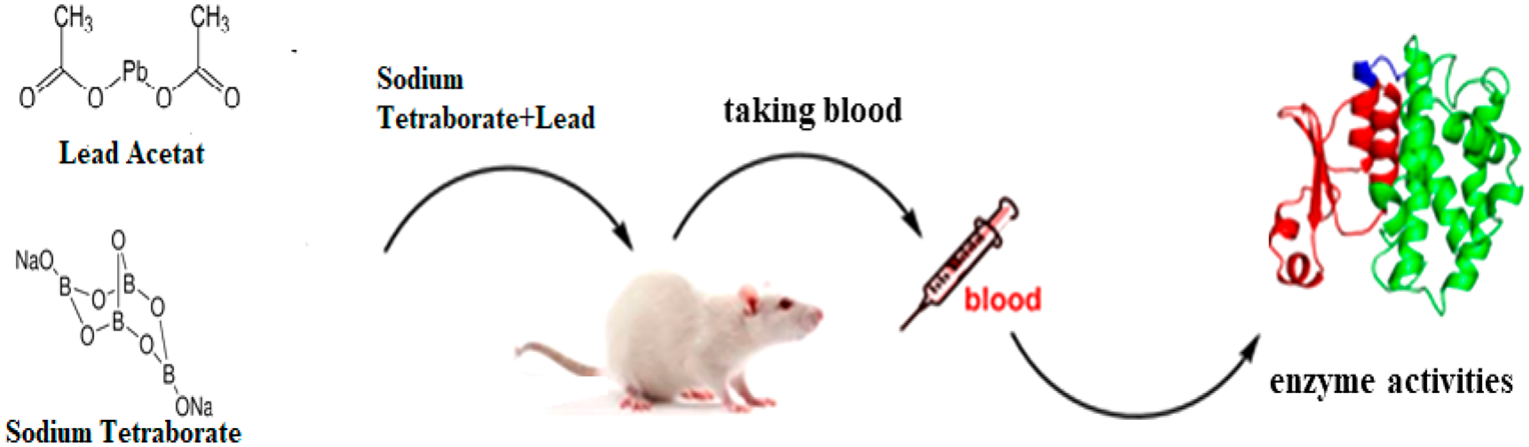

This study was planned to research the *in vivo* effects of lead (Pb) ions and sodium tetraborate (Na_2_B_4_O_7_) on G6PD and 6PGD, which are some of the enzymes of the pentose phosphate pathway, which carries vital importance for metabolism, and GR and GST, which are glutathione metabolism enzymes, and the *in vitro* effects of the same agents on the 6PGD enzyme. According to the *in vivo* analysis results, in comparison to the control group, the rat liver G6PD (*p* < 0.05), and 6PGD (*p* < 0.01) enzyme activities in the Na_2_B_4_O_7_ group were significantly lower. In addition, GR and GST enzyme activities were insignificantly lower in the Na_2_B_4_O_7_ group compared to the control group (*p* > 0.05). The Pb group had lower G6PD and 6PGD enzyme activity levels and higher GR and GST enzyme activity levels compared to the control group, while these changes did not reach statistical significance (*p* > 0.05). In the *in vitro* analyses of the effects of Pb ions on the 6PGD enzyme that was purified out of rat liver with the 2′,5′-ADP-Sepharose 4B affinity chromatography method, it was determined that Pb ions (200–1200 μM) increased the rat liver 6PGD enzyme activity levels by 33%. On the other hand Na_2_B_4_O_7_ was not significantly effective on 6PGD activity. These results will also contribute to future studies in understanding the physiopathology of the states triggered by Pb ions and sodium tetraborate (Na_2_B_4_O_7_).

## 1 Introduction

Lead (Pb) is a glossy, bluish-silver heavy metal that is naturally found in the environment.^1^ Pb, one of the metals that have been known for the longest time, is a prevalent and permanent source of environmental toxicity, and although Pb poisoning continues to be a health threat.^2^ it is still being used in several developing countries due to its unique physical and chemical properties.^3^ According to the World Health Organization, contact with toxic materials such as Pb and various other metals can promote the emergence or exacerbation of pathologic processes.^4^ Pb has been reported to have several toxic effects such as hematological,^5^ immunological,^6^ renal,^7,8^ hepatic,^9,10^ and reproductive dysfunction^11,12^ effects. One of the most significant proposed mechanisms of Pb toxicity is the disruption of the oxidant–antioxidant balance which leads to oxidative stress in cells.^13^ Boron (B), whose atomic number is 5, is a metalloid belonging to Group IIIA of the periodic table, and it is used in the forms of borax, colemanite, boronated-calcite, and boric acid.^14^ B is a trace element for plants, humans, and other animals.^15^ Boron is an element that is found in nature.^16^ It is found at high concentrations in sedimentary rocks, seawater, coals, and soils.^17^ Many studies have reported that boron has antigenotoxic,^15^ antioxidant,^18^ and antitumor^19^ properties. Glucose 6-phosphate dehydrogenase (d-glucose 6-phosphate: NADP^+^ oxidoreductase, EC 1.1.1.49; G6PD) is a highly important enzyme in the catalysis of the initial stage of the pentose phosphate metabolic pathway.^20,21^ This enzyme provides the largest amount of the NADPH for cells via the oxidation of glucose-6-phosphate into 6-phosphogluconate. NADPH has a considerable position in many functions in the body and a critical role in the antioxidant system, while it also defends the body against substances that induce oxidative stress.^22,23^ 6-Phosphogluconate dehydrogenase (6PGD, EC 1.1.1.44) is a highly prominent enzyme of the pentose phosphate pathway (PPP) that transforms 6-phosphogluconic acid (6PGA) into ribulose 5-phosphate and CO_2_ by the synthesis of NADPH.^24−26^ 6PGD is characterized by kinetic and acidic chemical mechanisms, and this reaction produces NADPH, which protects the cell against oxidative agents, by producing reduced glutathione.^27^ Glutathione reductase (GR; NADPH: glutathione reductase, EC 1.6.4.2), which is a flavoenzyme, is a significant biomolecule in terms of catalyzing the transformation of oxidized glutathione into its reduced form.^28^ By preserving a high GSH/GSSG ratio, GR not only scavenges free radicals and reactive oxygen species but also makes many vital functions of the cell such as the detoxification of protein and DNA biosynthesis possible.^29−31^ Glutathione *S*-transferase (GST) is an enzyme with multiple functions that has a significant part in the metabolic pathway of detoxification through the catalysis of the initial stage of the synthesis of water-soluble mercapturic acids, and it is found in rats, humans, and mice, particularly in liver tissue.^32,33^ In the literature review, no study on the effects of lead ions and sodium tetraborate on regulatory enzymes in the pentose phosphate pathway, which carries vital importance for metabolism, and glutathione antioxidant system enzymes was found. For this reason, this study was organized for investigating the *in vivo* effects of Pb ions and Na_2_B_4_O_7_ on the G6PD, 6PGD, GR, and GST enzymes and their *in vitro* effects on the 6PGD enzyme.

## 2 Materials and Methods

### 2.1 Chemicals

Pb, Na_2_B_4_O_7_, G6PD, 6PGD, Tris, NADP^+^, protein assay reagent, NADPH, DTNB, and standard serum albumin were obtained from Pharmacia (New Jersey, USA), and 2′,5′-ADP Sepharose-4B was purchased from Sigma. All other chemicals, which were of analytical purity, were purchased from Merck or Sigma (Germany).

### 2.2 Preparation of Crude Extract

The collected liver tissues were divided into small bits and pulverized using liquid nitrogen. Next, to the liver tissue specimens, a solution containing 1 mM EDTA + 2 mM DDT + 20 mM Tris–HCl (pH 7.5) was added. The buffer-containing mixture was centrifuged at 12,000 rpm for approximately 20 min at 4 °C. The supernatant was used in the experiments.^34,35^

### 2.3 Purification of 6PGD Enzyme

The 2′,5′-ADP-Sepharose 4B column was prepared based on a previously reported method. The supernatant was introduced to the column with 10 mL of column material. The column was subjected to washing using 50 mM phosphate buffer (1 mM DTT, 1 mM EDTA, pH 7.35). The 6PGD enzyme was separated using 80 mM phosphate +80 mM KCl + 0.5 mM NADP^+^ + 1 mM EDTA at pH 7.85. All steps were followed at a temperature of 4 °C.^36,37^

### 2.4 Determination of Enzyme Activity

The activities of the G6PD and 6PGD enzymes are determined spectrophotometrically based on the absorbance of NADPH at 340 nm.^38,39^ We utilized the technique proposed by Carlberg and Mannervik to measure GR enzyme activity levels. Enzyme activity is indicated by a reduction in NADPH in the reaction that the GR enzyme catalyzes. To determine enzyme activity, this reduction was read at 340 nm with a spectrophotometer.^40^ The quantification of the activity levels of the GST enzyme was dependent on the transformation of the CDNB substrate into the DNB-SG product by the GST enzyme when glutathione was present and the display of maximal absorbance by this substrate at a wavelength of 340 nm.^41,42^ Results were given as enzyme units (U/mg prot).

### 2.5 Protein Determination

The quantitative amounts of protein were determined spectrophotometrically at 595 nm according to the Bradford method. The standard curve was created using bovine serum albumin.^43^

### 2.6 In Vitro Effects of Sodium Tetraborate and Lead Acetate

To analyze the effects of Na_2_B_4_O_7_ and Pb on the enzyme activity of 6PGD, six Na_2_B_4_O_7_ concentrations (0.05, 0.1, 0.5, 1, 2.5, and 5 mM) and six different Pb concentrations (0, 150, 300, 600, 900, and 1200 μM) were separately introduced to tubes that included the purified enzyme. The IC_50_ value (concentration of the inhibitor reducing the total enzyme activity by half) and % activity – [I] curves were plotted and examined in the MS Office Excel program.^44^

### 2.7 In Vivo Effects of Sodium Tetraborate and Lead Acetate

Wistar Albino male rats weighing 200–300 g were used in the study. The rats were fed ad libitum. They were housed under a constant photoperiod with normal amounts of light and dark (12L:12D). During the experiments, the ambient temperature and relative humidity were set at 20 ± 3 °C and 40–60%, respectively. All experiments were carried out by complying with the ethical rules stated in the Guide for the Care and Use of Laboratory Animals. The protocol of the study was authorized by the Animal Experiments Ethics Committee of Bingöl University (BUHADEK:18.05.2021-2021/02). Twenty-four rats were divided into four groups (*n* = 6 each): Control (0.5 mL, i.p. isotonic solution), Pb (50 mg/kg/day i.p., Merck, USA),^45^ Na_2_B_4_O_7_ (4.0 mg/kg/day oral) (Sigma, USA),^46^ and Pb + Na_2_B_4_O_7_. After the fifth day, anesthesia was induced in the rats using 60 mg/kg i.p. ketamine hydrochloride and 10 mg/kg i.p. xylazine. Liver tissues were removed by median laparotomy, washed with phosphate-buffered saline (PBS), and kept in a deep freezer (−80 °C) until the analyses.

## 3 Analysis of Kinetic Data

The data are presented as mean ± standard deviation. Shapiro–Wilk and Levene’s tests were applied to test the normality and homogeneity of the data, respectively. The in vivo influence of Pb and Na_2_B_4_O_7_ on the G6PD, 6PGD, GR, and GST enzyme activities of groups was analyzed using the method of one-way analysis of variance (ANOVA), after which Tukey’s multiple comparisons test was performed. The analyses were performed using the SPSS statistics program (22.0, Chicago, IL, USA) and GraphPad Prism for Windows ver. 5.0 program (GraphPad software Inc., San Diego, CA, USA). For the in vitro analyses, Microsoft Office Excel 2010 was utilized. For the evaluation of the results, *p* < 0.05 was accepted as statistically significant.

## 4 Results and Discussion

**Figure 1:**
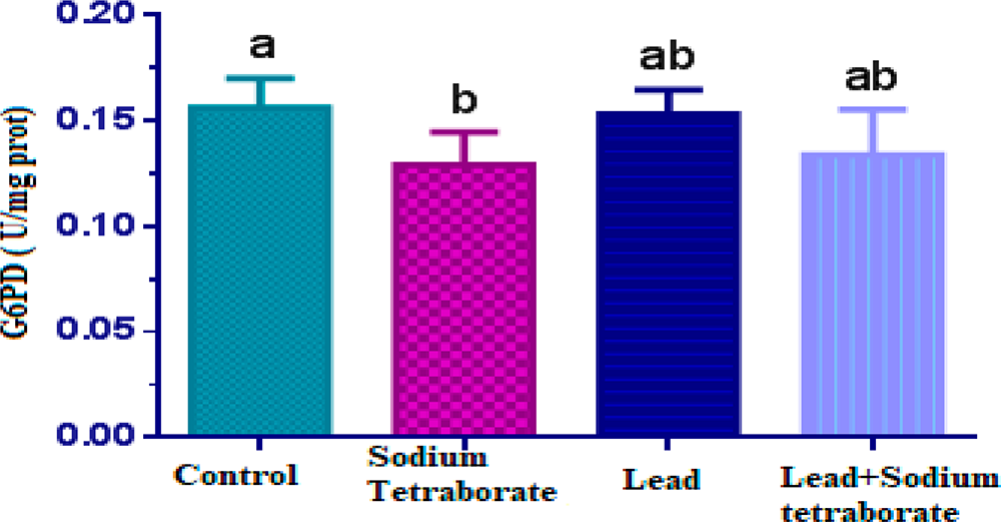
*In vivo* effects of lead (Pb) and sodium tetraborate (Na_2_B_4_O_7_) on rat liver G6PD (U/mg prot) enzyme activity. In parts a and b, there is a significant difference between groups with different letters. *p* < 0.05.

**Figure 2:**
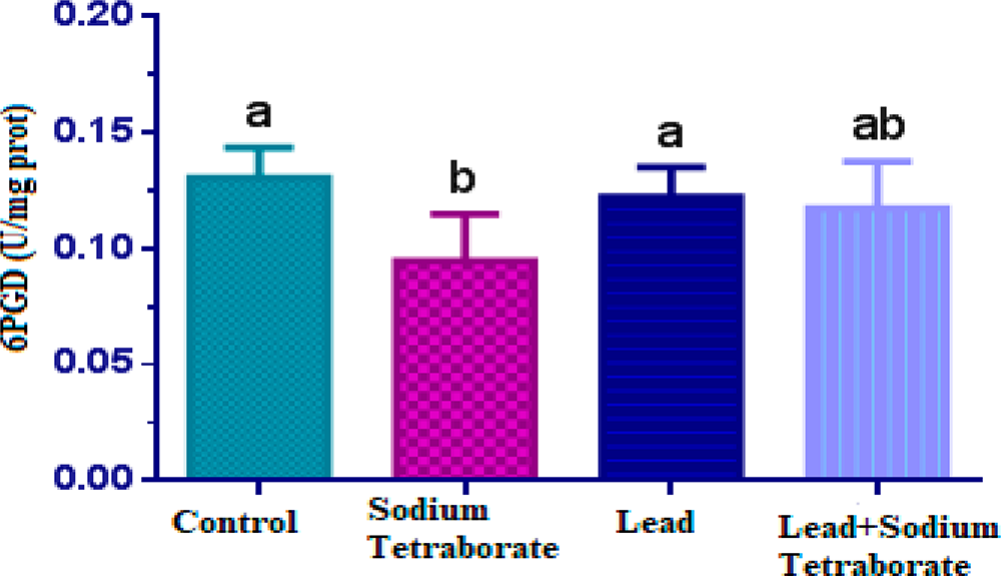
*In vivo* effects of lead (Pb) and sodium tetraborate (Na_2_B_4_O_7_) on rat liver 6PGD (U/mg prot) enzyme activity. In parts a and b, there is a significant difference between groups with different letters. *p* < 0.01

**Figure 3:**
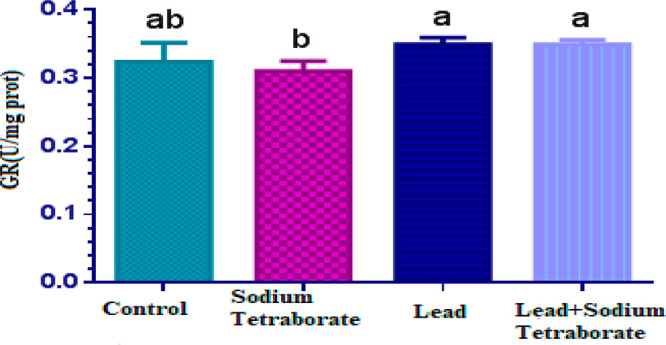
*In vivo* effects of lead (Pb) and sodium tetraborate (Na_2_B_4_O_7_) on rat liver GR (U/mg prot) enzyme activity. In parts a and b, there is a significant difference between groups with different letters. *p* < 0.01.

**Figure 4:**
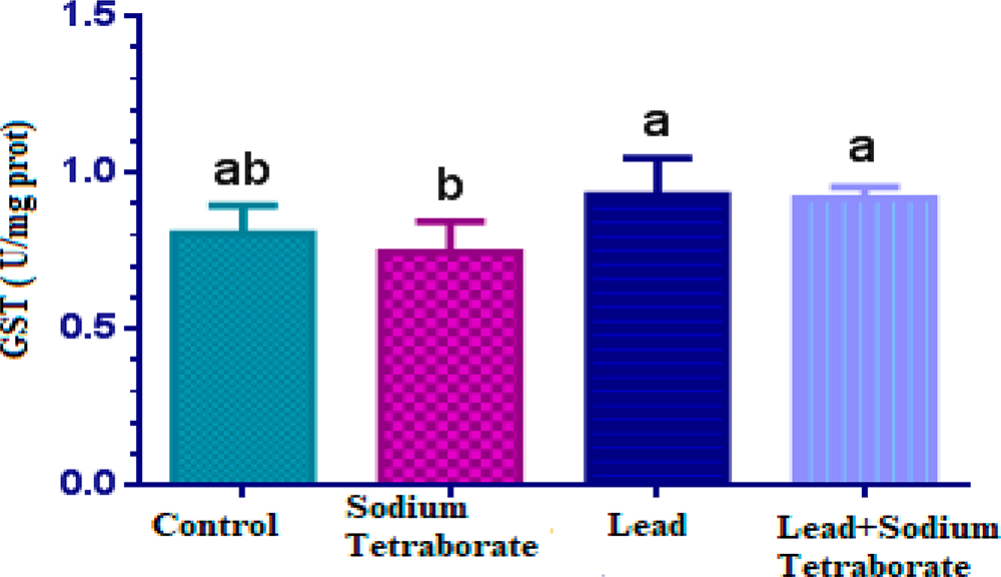
*In vivo* effects of lead (Pb) and sodium tetraborate (Na_2_B_4_O_7_) on rat liver GST(U/mg prot) enzyme activity. In parts a and b, there is a significant difference between groups with different letters. *p* < 0.01.

**Figure 5:**
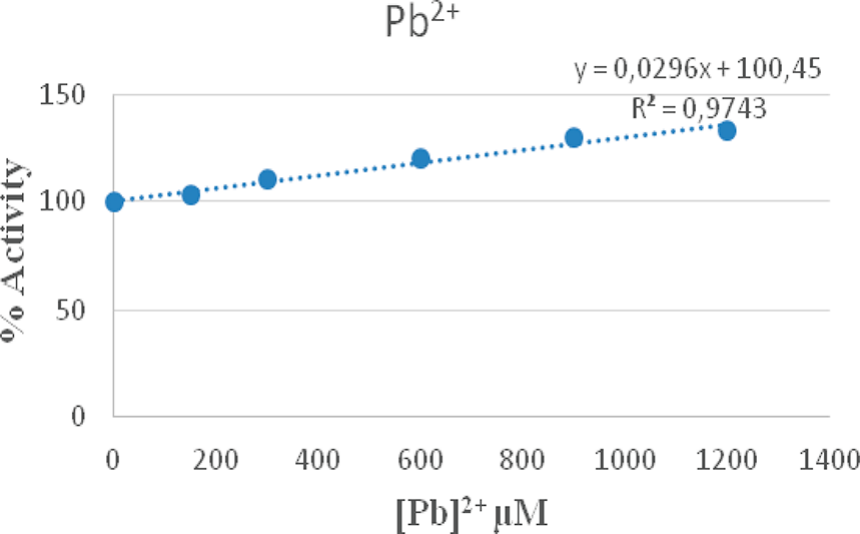
*In vitro* effects of lead (Pb) on rat liver 6PGD enzyme activity.

In the first part of the study, the *in vivo* effects of lead (Pb) ions and Na_2_B_4_O_7_ on G6PD, 6PGD, GR, and GST enzyme activities were investigated. The *in vivo* changes in the enzyme activity values in the lead (Pb), sodium tetraborate (Na_2_B_4_O_7_), and lead (Pb) + sodium tetraborate (Na_2_B_4_O_7_) groups in comparison to the control group are shown in the plots in Figures 1–5.

In comparison to the levels in the control group, the activity levels of G6PD in the Na_2_B_4_O_7_ group were significantly lower (*p* < 0.05). The activity levels of G6PD in the Pb group were lower compared to those in the control group, but this difference did not reach statistical significance (*p* > 0.05). The G6PD enzyme activity levels in the Pb + Na_2_B_4_O_7_ group were higher than those in the Na_2_B_4_O_7_ group and closer to those in the control group, and they were lower in comparison to the values in the control group to a statistically insignificant extent (*p* > 0.05) (Figure 1).

In comparison to the levels in the control group, the activity levels of 6PGD in the Na_2_B_4_O_7_ group were significantly lower (*p* < 0.01). The activity levels of 6PGD in the Pb group were significantly higher compared to the values in the Na_2_B_4_O_7_ group (*p* < 0.01). The 6PGD enzyme activity levels in the Pb + Na_2_B_4_O_7_ group were higher in comparison to those in the Na_2_B_4_O_7_ (*p* > 0.05) (Figure 2).

In comparison to the control group, the GR enzyme activity levels in the Na_2_B_4_O_7_ group were lower by an insignificant difference (*p* > 0.05). The activity levels of GR in the Pb group were insignificantly higher compared to the activity levels identified in the control group (*p* > 0.05). The GR enzyme activity levels in the Pb+Na_2_B_4_O_7_ group were higher in comparison to the values in the Na_2_B_4_O_7_ group (*p* < 0.01) (Figure 3).

In comparison to the control group, the activity levels of GST in the Na_2_B_4_O_7_ group were lower to an insignificant degree (*p* > 0.05). The activity levels of GST in the Pb group were insignificantly higher compared to those in the control group (*p* > 0.05). The GST enzyme activity measurements in the Pb + Na_2_B_4_O_7_ group were higher compared to the values in the Na_2_B_4_O_7_ group (*p* < 0.01) and closer to those in the Pb group (Figure 4).

At the subsequent stage of the study, the *in vitro* effects of Pb ions and Na_2_B_4_O_7_ on the 6PGD enzyme that was obtained from rat liver by using the 2′,5′-ADP-Sepharose 4B affinity chromatography method were investigated. The results of the analyses showed that Pb ions (200–1200 μM) increased the rat liver 6PGD enzyme activity levels by 33%. On the other hand Na_2_B_4_O_7_ did not affect 6PGD enzyme activity to a significant extent (Figure 5).

Pb is an element that is highly toxic for biological systems and is encountered frequently as an environmental and industrial pollutant. Although the toxicity of Pb ions in metabolism has not been completely explained yet, existing data show that exposure to Pb ions increases free oxygen radicals excessively, and thus, cellular antioxidant capacity is substantially affected by this exposure. It is known that an imbalance between oxidant and antioxidant systems leads to severe effects including membrane, DNA, and protein damage that extends to tissues or even systems.^47^ Scientific studies and clinical experiences so far have frequently revealed that Pb ions lead to dysfunctions in various tissues and organs. It was stated that in laboratory animals and humans, Pb led to high rates of physiological and biochemical dysfunctions mainly in the central and peripheral nervous systems, as well as other systems including the hematopoietic system, cardiovascular system, reproductive system, kidneys, and the liver.^48^ Moreover, it was frequently observed that Pb affected erythrocyte membranes, and it was suggested that there could be a relationship between changes in erythrocyte membranes and Pb-induced anemia.^49^ The liver is the main organ that takes part in the detoxification process, and it is one of the target organs that are influenced by Pb toxicity as Pb accumulates in the liver.^50^ It is known that Pb leads to oxidative damage in the liver, brain, testes, and kidneys by increasing lipid peroxidation.^51^ Recent studies have shown that oxidative stress is one of the significant mechanisms of the toxic effects of Pb.^52−56^ Likewise, Tandon et al. (1997),^57^ El-Sayed et al. (1995),^58^ and Khan et al. (1993)^59^ reported their observations of liver dysfunctions following chronic exposure to Pb. A previous study demonstrated that acute exposure to Pb ions caused toxic effects in the blood, liver, and kidneys of adult Wistar rats. A disrupted redox profile was identified in the examined tissues of the rats.^60^ The effects of Pb-induced toxicity on liver, kidney, brain, and heart tissues were examined in Wistar rats by analyzing the activities of CAT, SOD, GST, GPx, and GR, which are significant enzymes of the antioxidant system that act as the frontline defense against oxidative damage. Inhibition was shown in the antioxidant enzyme activity levels of the rats that were exposed to Pb, and a significant decrease occurred in the glutathione levels of these rats. Additionally, lipid peroxidation, DNA fragmentation, and hematological parameters substantially changed in the rats that were administered Pb acetate compared to the controls.^2^ In another study where the effects of Pb were investigated, it was observed that Pb treatment reduced weight, levels of hematocrit, and blood δ-aminolaevulinic acid dehydratase (ALA-D) function and resulted in elevated Pb levels in blood and tissues, raised the lipid peroxidation levels in erythrocytes, plasma, and tissues, and caused protein oxidation in tissues. Furthermore, reductions were observed in superoxide dismutase (SOD) activity and catalase (CAT) activity in the liver.^61^ In a study that was performed to understand the biochemical mechanisms of Pb toxicity in the liver of rats, after the administration of Pb, a significant accumulation of Pb and increased lipid peroxidation were detected in the liver. It was reported that along with the increase in lipid peroxidation, glutathione reductase (GR) enzyme activity was significantly inhibited.^62^ In a different study, in which the effects of acute Pb acetate exposure on glutathione *S*-transferase (GST) subunit expressions and the quantities of reduced and oxidized glutathione (GSH) and malondialdehyde (MDA) in rat kidneys and livers were examined, Pb injection into the liver led to a reduction in GSH concentrations and an increase in the production of MDA, and the increased concentration of MDA results in increases in the activities of the GSTA1, GSTA2, GSTM1, and GSTM2 enzymes.^63^ It was reported that Pb administration caused inhibitions in the enzyme activity levels of testicular antioxidant enzymes including superoxide dismutase (SOD), catalase, glucose-6-phosphate dehydrogenase (G6PDH), and glutathione *S*-transferase (GST).^64^ Another study where the effects of Pb on the rat liver were examined revealed that Pb exposure increased the CAT enzyme activity of the liver.^65^ In a study where the effects of chronic Pb exposure on the oxidative stress statuses of the heart and liver were investigated in rats, it was determined that Pb exposure led to increased activity levels of SOD and CAT, which are important antioxidant enzymes, in the examined liver and heart tissues.^66^ Toz et al. reported that Pb given to rats caused an inhibition in G6PD enzyme activity levels, whereas chitosan reduced the degree of this inhibition and brought these enzyme activity levels closer to those of the control group.^67^ In a study of Pb-induced lipid peroxidation in the rat liver, the activity levels of GR, GST, and G6PD, which are significant hepatic metabolic enzymes, decreased in the group to which Pb was administered in comparison to the control group.^68^ Metabolic enzyme levels measured in the livers and kidneys of rats exposed to Pb were examined, and an increase in GR and GST activities in the kidneys was observed, while there was a decrease in liver enzyme activities.^69^ It was reported that Pb ions had an increasing effect on the activity levels of the TrxR enzyme purified from turkey liver tissue.^22,70^ It was determined that the liver GST and GR enzyme activity levels in rats exposed to Pb were lower in comparison to those in the control group.^71^ Boron compounds have well-defined biological effects and are described as compounds that can provide therapeutic benefits. Various studies have found that boron treatment leads to a decrease in liver GR, GST, and G6PD enzyme activity levels in rats compared to controls.^72^ A different study examined the effects of Na_2_B_4_O_7_ on erythrocyte superoxide dismutase (SOD), catalase (CAT) glutathione reductase (GR), glutathione *S*-transferase (GST), and glucose-6-phosphate dehydrogenase (G6PGD) enzyme activity levels and revealed that Na_2_B_4_O_7_ did not have any inhibition or activation effects on blood samples.^73^ In our study, in comparison to the control group, the rat liver activity levels of the G6PD and 6PGD enzymes in the Na_2_B_4_O_7_ group were significantly lower, while the activity levels of GR and GST in the Na_2_B_4_O_7_ group were also lower, albeit not significantly. The Pb group had lower G6PD and 6PGD enzyme activity levels and higher GR and GST enzyme activity levels in comparison to the control group, while these changes did not reach a statistically significant extent. In the *in vitro* analyses of the effects of Pb ions on the 6PGD enzyme that was obtained from rat liver using the 2′,5′-ADP-Sepharose 4B affinity chromatography method, it was determined that Pb ions (200–1200 μM) increased the rat liver 6PGD enzyme activity levels by 33%. Na_2_B_4_O_7_, on the other hand, was not significantly effective on the activity levels of the 6PGD enzyme. It may be stated that the results of our study were in agreement with the results of previous studies in the relevant literature.

## 5 Conclusion

As a result in this study; Effects of sodium tetraborate and Pb (lead) on some metabolic enzyme activities to rat liver cells under in vivo and in vitro conditions were studied. According to the *in vivo* analysis results, in comparison to the control group, the rat liver G6PD (*p* < 0.05), and 6PGD (*p* < 0.01) enzyme activities in the Na_2_B_4_O_7_ group were significantly lower. In addition, GR and GST enzyme activities were insignificantly lower in the Na_2_B_4_O_7_ group compared to the control group (*p* > 0.05). The Pb group had lower G6PD and 6PGD enzyme activity levels and higher GR and GST enzyme activity levels compared to the control group, while these changes did not reach statistical significance (*p* > 0.05). In the *in vitro* analyses of the effects of Pb ions on the 6PGD enzyme that was purified out of rat liver with the 2′,5′-ADP-Sepharose 4B affinity chromatography method, it was determined that Pb ions (200–1200 μM) increased the rat liver 6PGD enzyme activity levels by 33%. On the other hand Na_2_B_4_O_7_ was not significantly effective on 6PGD activity. These results will also contribute to future studies in understanding the physiopathology of the states triggered by Pb ions and sodium tetraborate (Na_2_B_4_O_7_).

## Notes

**Ethical Considerations.** This study was approved by the Local Ethics Committee (BUHADEK:18.05.2021-2021/02) for Animal Experiments.

## Abbreviations

DTNB: 5,5-dithiobis(2-nitrobenzoic acid)
DDT: dichlorodiphenyltrichloroethane
CDNB: 1-chloro-2,4-dinitrobenzene
EDTA: Ethylenediaminetetraacetic acid
G6PD: glucose 6-phosphate dehydrogenase
6PGD: 6-phosphogluconate dehydrogenase
GR: glutathione reductase
GST: glutathione S-transferase
Pb: lead
NADP^+^: nicotinamide adenine dinucleotide phosphate
NADPH: nicotinamide adenine dinucleotide phosphate oxidase
Na_2_B_4_O_7_: sodium tetraborate
